# Two-stage repair in hypospadias

**DOI:** 10.4103/0970-1591.40620

**Published:** 2008

**Authors:** K. N. Haxhirexha, M. Castagnetti, W. Rigamonti, G. A. Manzoni

**Affiliations:** Department of Surgery, Hospital Center Tetovo, Tetovo, Macedonia; 1 Pediatric Urology Unit, Department of Urology, University Hospital of Padova, Padua; 2 Department of Urology and Section of Pediatric Urology - Ospedale di Circolo e Fondazione Macchi, Varese, Italy

**Keywords:** Balanitis xerotic obliterans, buccal mucosa, hypospadias, hypospadias cripple, staged repair

## Abstract

We provide the reader with a nonsystematic review concerning the use of the two-stage approach in hypospadias repairs. A one-stage approach using the tubularized incised plate urethroplasty is a well-standardized approach for the most cases of hypospadias. Nevertheless, in some primary severe cases, in most hypospadias failures and in selected patients with balanitis xerotica obliterans a two-stage approach is preferable. During the first stage the penis is straightened, if necessary and the urethral plate is substituted with a graft of either genital (prepuce) or extragenital origin (oral mucosa or postauricular skin). During the second stage, performed around 6 months later, urethroplasty is accomplished by graft tubulization. Graft take is generally excellent, with only few cases requiring an additional inlay patch at second stage due to graft contracture. A staged approach allows for both excellent cosmetic results and a low morbidity including an overall 6% fistula rate and 2% stricture rate. Complications usually occur in the first year after the second stage and are higher in secondary repairs. Complications tend to decrease as experience increases and use of additional waterproofing layers contributes to reduce the fistula rate significantly. Long-term cosmetic results are excellent, but voiding and ejaculatory problems may occur in as much as 40% of cases if a long urethral tube is constructed. The procedure has a step learning curve but because of its technical simplicity does not require to be confined only to highly specialized centers.

## INTRODUCTION

### Which cases should undergo a staged repair?

Despite a large number of techniques described, the history of hypospadias surgery seems to be a continuous revision of few themes with very few milestone innovations down the way.[1] One such recurring theme is the dispute between single vs. two-stage repairs. A two-stage repair was described in the early history of surgery and was subsequently abandoned as technical developments, such as the incorporation of the urethral plate in the repair and the use of dorsal plication for penile straightening, allowed a safe single-stage repair in most of the cases.[2,3]

Nowadays, there is a renewed interest in the two-stage repair, as it seems to be able to both reduce morbidity and improve cosmesis in the correction of the most severe forms of hypospadias.[4–8]

About 80-85% of hypospadias have a distal-shaft meatus and mild curvature, while the remaining present with a proximal meatus and severe curvature.[9]

The surgical strategy is aiming at multiple goals including orthoplasty (penile straightening), urethroplasty, glansplasty, meatoplasty, scrotoplasty, and skin coverage with circumcision or prepucial reconstruction.[10] All these steps can be performed in a single operation or staged. The technique should be tailored to each individual case, mainly based on the degree of curvature and the quality of the urethral plate.

Indeed, the vast majority of cases can be successfully treated in a single stage. Penile straightening is achieved by removal of the fibrous remnants on the ventral aspect of the penis and the urethra is created by tubularization of the urethral plate.[11] The latter can be performed after midline hinging of the plate (Snodgrass)[12] and with or without interposition of an in-lay graft between the margins of incision for augmentation (Snodgraft).[13]

**Figure 1:**
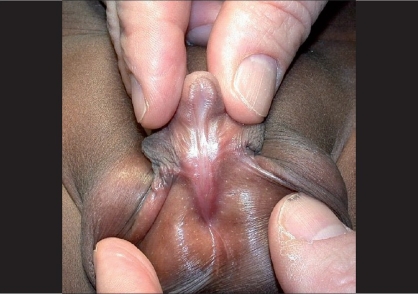
Primary case with underdeveloped urethral plate suitable for substitution

In a minority of cases, penile curvature is so severe to require a concomitant dorsal plication or a ventral corporal grafting to be corrected.[14] In some selected cases, even the urethral plate needs to be sectioned to achieve an adequate straightening. In others, although the urethral plate does not contribute to the curvature, may be underdeveloped and unsuitable to be incorporated in the urethroplasty [Figure 1]. All these cases, requiring a urethral plate substitution might benefit of a staged approach.[11]

Other major indications to urethral plate substitution and hence a potential staged approach, include patients with several previously failed procedures (hypospadias failures) and those with *balanitis xerotica obliterans* (BXO). The latter is one of the most important yet often unrecognized causes of late hypospadias failures. The condition cannot be cured by augmentation procedures, dilatations or endoscopic urethrotomies, which provide only temporary relief, while allowing insidious progression of disease.[15] Substitution with genital skin usually leads to re-stricture within a couple of years. Nongenital skin, such as postauricular Wolfe grafts, may allow more durable success, but even such procedures usually leads to re-stricture within 10 years. Substitution of the entire diseased segment with buccal mucosa appears as the only effective long-term solution in these patients.[15]

Single-stage repairs have also been reported in patients requiring urethral plate substitution, but, to accomplish the repair in a single stage, a tube needs to be constructed with a flap or a graft. These procedures are associated with a secondary surgery rate ranging from 30 to 90% without any apparent advantage of flaps over grafts.[16–19]

In conclusion, the decision as to proceed with a one-stage or a staged approach depends on several considerations including anatomical features, previous surgical history, and evidence of BXO. The final decision may often be taken only at surgery, especially in primary repairs in which the fate and the possibility to preserve the urethral plate can be assessed only after a sequential and standardized approach.

It should be noted that the term staged hypospadias repair encompasses, in the current literature, several different approaches including complete removal and substitution of the urethral plate[4–8] or only with transversal incision of the plate.[20] In this review, the term will be consistently used to describe a procedure including penile straightening and urethral plate substitution as first stage and urethroplasty as second stage.

## TECHNIQUE OF TWO-STAGE REPAIR

### Preparation of the penis and straightening

**Figure 2:**
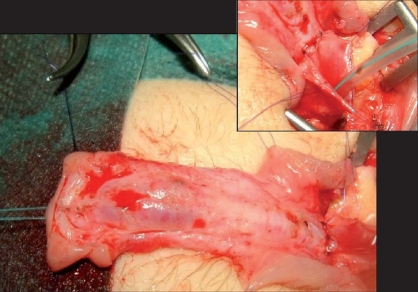
Penis after removal of the ventral remnants and deep midline incision of the glans. Inset: proximal urethrostomy

The procedure starts removing any fibrous tissue on the ventral aspect of the penis. In secondary cases, any poor quality distal urethra is removed too, whereas the urethral plate is initially spared in primary cases. Glans wings are developed performing a deep incision in the midline and lifting glanular tissue off the apex of the penile corpora laterally [Figure 2].

An erection test is performed at the end of this step. If curvature persists, we usually perform a dorsal plication. Therefore, we completely de-glove the penis, isolate the dorsal neurovascular bundle and plicate the tunica albuginea in the midline after creating multiple small transversal incisions in the portion we plicate.

This is the point in which, in primary cases, it is necessary to decide as to transect the urethral plate or to preserve it.

As an alternative to dorsal plication, some authors prefer to perform a ventral grafting to correct the curvature.[14] A third possibility is to achieve a ventral lengthening by performing multiple transversal incisions on the ventral aspect of the corpora and than to cover all with the free graft.[6] In both, the latter the urethral plate is discarded at the beginning of the procedure.

Next a proximal urethrostomy is performed. If the meatus is perineal, we usually try to advance it at the level of the penoscrotal junction. The spatulated meatus is anchored to the corpora cavernosa and adjacent skin [Figure 2, inset].

### Harvesting and preparation of the graft

**Figure 3:**
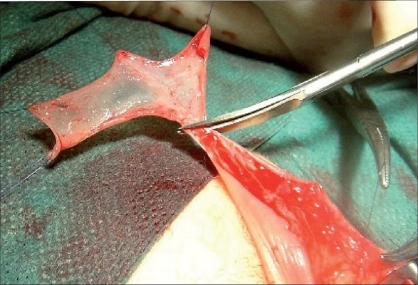
Preputial graft

The gap produced in the ventral aspect of the penis needs to be filled during the first stage usually with a free graft. The latter can be of genital or extragenital origin. The major genital harvesting site is the prepuce [Figure 3]. Some authors prefer to use preputial flaps rather than a graft. Byar's flaps can be transposed ventrally either to substitute entirely the plate or to fill the gap after urethral plate sectioning.[20,21]

The major extragenital harvesting site is instead the mouth.[18] Oral mucosa graft can be harvested from the inner cheek or from the lower lip and even from the tongue.[22,23] It should be noted, however, that according to standard and accepted dental terminology, the term buccal mucosa refers only to the oral mucosa overlying the inner cheek of the oral cavity.[24] An alternative extragenital harvesting site is the retroauricular region.[7,8]

The inner preputial layer whenever still available is, in our opinion, the graft of choice. Nevertheless, it is not available in most redo cases, may be fairly hypoplastic in some primary cases with severe hypospadias and should be avoided in all BXO cases.

**Figure 4:**
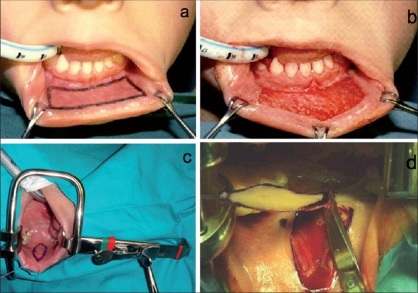
Graft harvesting from the lower lip (a and b) and from the inner check (c and d)

The oral mucosa is therefore the graft we most commonly use in staged reconstructions. We usually prefer to harvest the graft from the inner cheek, as the mucosa is more thick and solid [Figure 4]. The area to harvest should be outlined first with mandatory identification of Stensen's duct and then infiltrated with 1:100,000 epinephrine with bupivacaine. Infiltration makes subsequent dissection easier in order to minimize the amount of fat left on the under-surface and avoid dissection into the muscles. We usually close the donor site in the inner cheek with interrupted reabsorbable sutures. Fibrin glue can be used as an alternative.[6] If the graft is harvested from the lower lip, the donor site is always left open.[25]

**Figure 5:**
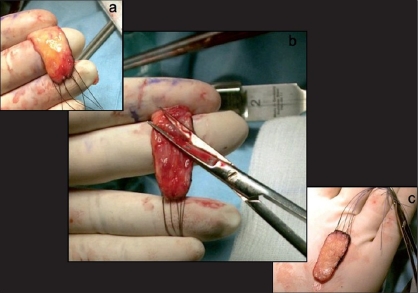
Buccal mucosa graft de-fatting

The under-surface of the graft is further defatted by placing the graft spread over a finger and removing the fat by sharp dissection with scissor [Figure 5].

Multiple passages in antibiotic solution are performed.

### Graft stabilization

**Figure 6:**
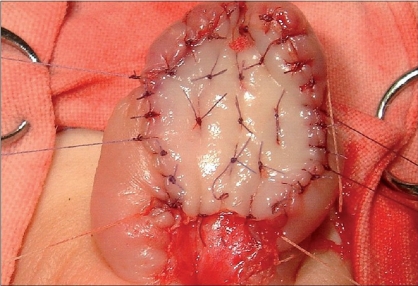
Final appearance of the graft at the end of first stage

The graft is then secured to the ventral aspect of the corpora. The perimeter is sewn first and then multiple quilting stitches are placed through the graft in multiple parallel rows in order to reduce the potential risk of an hematoma and graft loss [Figure 6].

A catheter is placed through the urethrostomy and fixed with a glandular stitch. Folded petroleum jelly gauze is placed between the catheter and the graft to keep the latter in place and to promote its healing process. A compressive dressing is applied while others prefer to use a tie-over dressing, consisting of a roll of petroleum jelly gauze held in place by sutures along the graft margins.[3,6]

Postoperatively, bed rest is recommended for 3-5 days depending on the extent of the graft and the age of the patient. Parenteral, large spectrum antibiotic coverage is administered for 3-5 days. The catheter is usually removed after 5-7 days.

### Second stage

**Figure 7:**
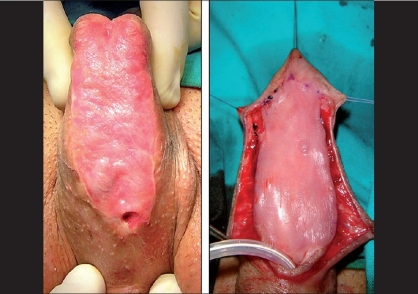
Second stage

**Figure 8:**
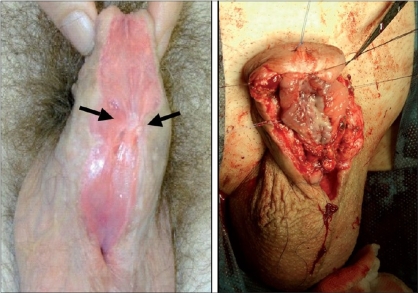
Graft contracture

**Figure 9:**
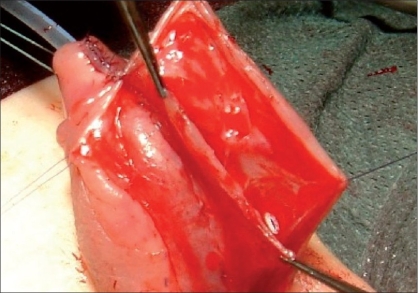
Second layer for urethroplasty coverage

The second stage, consisting of the tubularization of the graft [Figure 7], is generally planned after 6 months.[26] In this interval, graft shrinkage may occur in nearly 20% of cases. This should indeed be taken into consideration while harvesting. If excessive shrinkage occurs between procedures [Figure 8] the graft can be re-augmented during the second stage by placing a new free graft as in-lay after midline hinging.[6] Glansplasty is usually easily performed and allows for a deep placement of the neo-meatus and creation of a slit-like meatus. Additional waterproofing layers are recommended whenever possible [Figure 9]. In complex redo cases, a tunica vaginalis flap is often the only possible site to mobilize such an additional layer.

**Figure 10:**
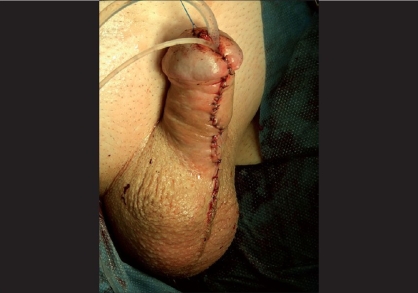
Final appearance

Skin coverage should ideally be performed by midline approximation of the skin with sub-epithelial interrupted stitches [Figure 10]. Skin coverage can, however, be sometimes quite difficult and may require fantasy.

After this stage we usually leave an indwelling trans-urethral catheter and a supra-pubic diversion. The former is removed after 7 days, while the latter after one further week during which it is kept closed, if no problems occur.

## OUTCOME OF STAGED HYPOSPADIAS REPAIR

Several variables should be considered in order to evaluate the success of a two-stage hypospadias repair. These include donor site morbidity, graft take after first stage, complications of urethroplasty (recipient site morbidity), and finally both the functional and cosmetic outcomes.

Regarding donor site morbidity, usually oral bleeding is not a problem in these patients. Oral numbness and tightness of the mouth are instead quite common especially in the immediate postoperative period.[27–29] Oral numbness is considered to be due to a neuropathy of the nerve and has been reported to last longer and to be more common a cause of dissatisfaction in patients undergoing graft harvesting from the lower lip.[28] Oral numbness and tightness of the mouth do not usually interfere with resumption of drink and food intake, which can usually be started immediately after surgery. In around 5% of cases, however, feeding can remain troublesome even 6 months after surgery.[28] Problems of permanent scarring at the donor site are quite rare.

Once the graft is quilted onto the corpora, the first issue is graft take. Mokhless *et al.* conducted histologic studies on the grafted mucosa and found that the free graft showed excellent uptake within 5 days. At 6 months, the buccal mucosa was well-vascularized and pliable displaying epithelial hyperplasia with mild and focal keratinization.[26] As mentioned above, a retraction of about 20% of the grafted area usually occurs between stages; therefore, harvesting a graft slightly larger than required is recommended. Excessive retraction may require augmentation during second stage, but no figures have been published regarding the occurrence of such a complication. A group from London reported a complete take of the graft in 100% (62 out of 62) of primary repairs and 95% (40 out of 42) of secondary cases.[7,8] Snodgrass and Elmore reported a slightly lower success rate with an 88% rate of complete graft take in secondary repairs.[6] In the remaining cases instead graft patching was required before tubularization due to focal scarring or graft contracture.[6]

As for hypospadias surgery, also in the two-stage repair most of the urethroplasty complications tend to occur in the first 6-12 months after the second stage,[30] but for cases with BXO in whom the occurrence of progressive retraction of the tube may result over years.[15] In oral mucosa repairs, recipient site success seems overall to be significantly higher when the graft is harvested form the buccal rather then the labial site.[24] As for all other techniques, major complications of the staged repair include fistula formation, urethral stricture, and meatal stenosis.

Bracka in his series of more than 600 procedures reported a gross fistula rate of 5.7%.[4] Most occurred during the learning curve and this was also confirmed by Hensle *et al.* which reported a complication rate dropping from 60% during the first 3 years of their experience to 19% in the last 7 years.[30]

Bracka also reported a fistula rate much higher in secondary (10.5%) than primary repairs (3%).[4] The adjunct of a waterproofing layer seems to be crucial to reduce the fistula rate. Accordingly, Telfer *et al.* reported a 63% fistula rate in cases operated without and a 4.5% rate in those operated with such an additional layer.[31]

Stricture rate was 7% in Bracka's experience.[4] Two-third of these, however, proved to be due to the presence of BXO leaving only a 2% rate of strictures related to surgery.[4] Of the latter, 70% were successfully treated by dilatation and 30% required a surgical revision.[4]

Partial glans dehiscence is a recognized complication of the two-stage repair and is reported in almost all series with an incidence ranging between 5 and 25%.[5–8]

One of the major advantages of staged repairs is the possibility to achieve a good cosmetic result with placement of the urethra deep in the glans and creation of a natural slit-like meatus. Accordingly, most of the series report an excellent cosmetic results and patient satisfaction.[28] It should be noted, however, that Bracka reported a 5.5% of cases requiring additional revisional surgery after second-stage for cosmetic adjustments.[4]

Data on the long-term outcome of staged repairs are lacking. Lam *et al.* reported the long-term outcome in patients undergoing a modified Belt-Fuqua repair.[32] All had satisfactory cosmetic outcome with normal meatal position, normal glanular anatomy, well-defined coronal sulcus, normal cylindrical shaft, and well-defined penoscrotal junction. None had recurring curvature. Functional outcome was instead less satisfactory. Some 40% had minor spraying of stream, 40% needed to milk their urethra after voiding, and 43% had to milk the urethra to ejaculate.[32]

In his experience, Bracka reported a 40% urine dribbling rate, 33% of his patients could only have a dribble of ejaculate and 45% had whole retained ejaculate.[33]

These data seem to suggest that voiding and ejaculatory problems do occur after any staged repair and are probably related to the construction of a long urethra without the normal support of spongiosum tissue.

It might be questioned as to whether the two-stage repair, being a complex approach, should be left to experienced surgeons with long-lasting experience in the field and devoted to hypospadias surgery. Evidence suggest that also this approach has a step learning curve, which can create problems at the beginning; however, it can be accomplished even by surgeons without a specific interest in hypospadias surgery and by trainees.[34,35] Titley and Bracka performed a 5-year audit of the trainees performing two-stage repairs. Complications rates for the first stage were similar among consultants (4.5%), supervised trainees (5.3%), and unsupervised trainees (7.3%), whereas unsupervised trainees had a much higher complication rate for the second stage (29.6%) compared to consultants (3.0%) and supervised trainees (5.3%). Therefore, it seems that this staged approach should initially be performed under supervision of an experienced surgeon and that the second stage is the more critical stage.[35]

## PERSONAL EXPERIENCE

Between 2002 and 2007, 36 patients underwent a staged oral mucosa redo hypospadias repair at our Institutions.

Donor site morbidity included delayed resumption of food in one case and donor site scarring in another. In both cases, the graft was harvested from the inner cheek.

Graft take was complete in all patients with normal healing in 32 patients (89%), whereas in the remaining four another patch was required, because of partial contraction/scarring, in order to accomplish the second stage.

Overall complication rate of second stage was 8.3% (3 out of 36 patents) with all the complications presenting within 6 months from surgery. A small single fistula developed in two patients (5.5%) and both were successfully treated with a multilayer repair. One further case experienced a partial glans dehiscence and required a revision glansplasty. No meatal stenosis, urethral stricture, or diverticula formation were recorded.

All cases had a normal-looking penis with a slit-like meatus at last follow-up.

## CONCLUSIONS

In summary, the staged repair is a safe and reliable approach in selected patients in whom the urethral plate cannot be incorporated in the repair and thus requires substitution. These include both primary hypospadias with severe curvature, complex redo cases, and cases with BXO. The graft for urethral plate substitution can be harvested from genital or extragenital sites. Preputial grafts are the grafts of choice in primary repairs, whereas buccal mucosa is the graft of choice in secondary repairs and in patients with BXO. The second stage should be performed usually nearly 6 months after the first stage and should be completed with additional waterproofing layers. The procedure can be accomplished with a low-complication rate, 6% fistula rate and 2% stricture rate, and with a good final cosmetic results. Patients should be informed that construction of a long urethra can be associated with long-term voiding and ejaculatory problems in as much as 40% of cases. The procedure has a step learning curve but does not require being confined only to highly specialized centers.
